# Methodological Problems in fMRI Studies on Acupuncture: A Critical Review with Special Emphasis on Visual and Auditory Cortex Activations

**DOI:** 10.1093/ecam/nep154

**Published:** 2011-02-13

**Authors:** Florian Beissner, Christian Henke

**Affiliations:** ^1^Brain Imaging Center, Goethe-University, Frankfurt, Neuroradiology, Schleusenweg 2-16, 60528 Frankfurt, Germany; ^2^Institute of Neuroradiology, Goethe-University, Frankfurt, Germany; ^3^Max-Planck-Institute of Biophysics, Goethe-University, Frankfurt, Germany; ^4^Department of Neurology, Goethe-University, Frankfurt, Germany

## Abstract

Functional magnetic resonance imaging (fMRI) has been used for more than a decade to investigate possible supraspinal mechanisms of acupuncture stimulation. More than 60 studies and several review articles have been published on the topic. However, till now some acupuncture-fMRI studies have not adopted all methodological standards applied to most other fMRI studies. In this critical review, we comment on some of the problems including the choice of baseline, interpretation of deactivations, attention control and implications of different group statistics. We illustrate the possible impact of these problems by focussing on some early findings, namely activations of visual and auditory cortical areas, when acupoints were stimulated that are believed to have a therapeutic effect on vision or hearing in traditional Chinese medicine. While we are far from questioning the validity of using fMRI for the study of acupuncture effects, we think that activations reported by some of these studies were probably not a direct result of acupuncture stimulation but rather attributable to one or more of the methodological problems covered here. Finally, we try to offer solutions for these problems where possible.

## 1. Introduction

**Figure 1:**
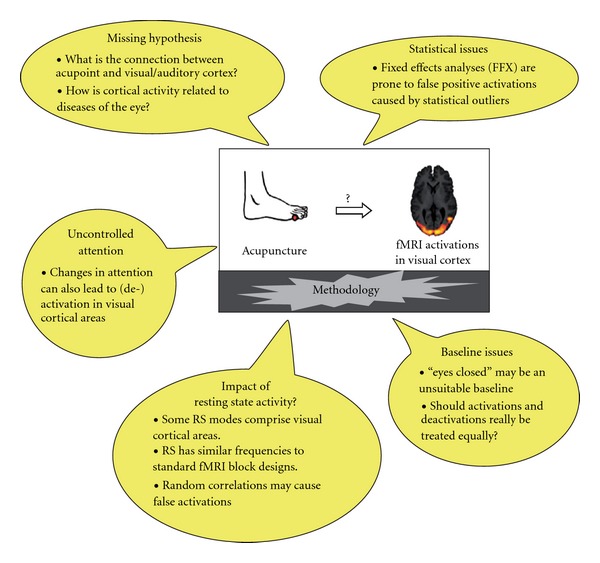
Possible explanations other than acupuncture for activations of visual cortical areas found in acupuncture-fMRI studies. Only visual areas have been covered here, although the same reasoning applies to auditory cortical areas.

Since the late 1990s, functional magnetic resonance imaging (fMRI) has been used to investigate the underlying mechanisms of the Chinese medical therapy of acupuncture. After the proposal of the idea [1] the groups of Hui and Cho were the first to show cortical activations associated with acupuncture stimulation using fMRI [2, 3]. The study by Cho et al. [4] is still referenced in the literature as a breakthrough in acupuncture research. However, this paper was later retracted by five of the eight authors. In the meantime, more than 60 studies have been published that tried to elucidate the underlying mechanisms of acupuncture by means of fMRI. However, researchers working in this field are still facing a number of unsolved problems and up to now some acupuncture-fMRI studies have not adopted all methodological standards applied to most other fMRI studies (Figure 1). In this critical review we would like to comment on some of these problems and try to offer solutions, where possible. We have illustrated the possible impact of these problems by focussing on some early findings that have sparked a lot of interest, namely activations of visual and auditory cortical areas under stimulation of acupoints that are believed to have a therapeutic effect on vision or hearing from the viewpoint of traditional Chinese medicine (TCM). We will show that, there exist several mechanisms that can cause such activations independent of the actual acupuncture stimulation. We will not try to review acupuncture-fMRI studies as a whole since there are excellent and up-to-date reviews available [5–7]. Neither will we cover general problems of acupuncture research like the question of placebo and sham interventions [8] or differences between stimulation methods [9].

## 2. Identification of Relevant Literature

Relevant publications for this review were identified by a systematic search of PubMed using the terms “acupuncture” and “fMRI” and of reference lists of retrieved articles. After discarding unrelated publications we checked all remaining 67 publications (see supplementary material for a complete list) for their choice of acupoints and reported cortical activations. We identified eight studies that investigated the cortical effect of vision-related acupoints [3, 10–16] and two studies that did the same for hearing-related acupoints [14, 17].  Table 1 gives an overview of all nine studies covered in this review.

## 3. General Observations

### 3.1. A Comment on Possible Underlying Mechanisms

The putatively logical relationship between the therapeutic potential of vision- or hearing-related acupoints and activations of cortical areas that process these sensations may be one reason for the popularity of the results. However, an anatomical link between the lateral aspect of the foot (acupoints of the BL conduit) and the visual cortex could not be demonstrated so far. The same holds true for the auditory points used. However, such a relationship may exist on the cortical level. As several studies have shown, sensory stimulation can activate both visual and auditory cortices [18–20]. The strong sensory component of acupuncture is self-evident. However, its relation to the underlying mechanisms of acupuncture is yet to be proven. Furthermore, if we take a standard acupuncture book [21] and look up common indications for the two most often used points BL60 and BL67, we mainly find that the eye-related symptoms (“redness, pain and swelling of the eyes”, “eye pain”, “pain of the inner canthus”, etc.) have nothing to do with the visual cortex. Other indications such as “visual dizziness” and “superficial visual obstruction” also have little or no connection to the function of the visual cortex. Similar arguments apply to auditory acupoints also. Thus, if there is a measurable effect of these acupoints on vision or hearing, it seems unlikely that this effect will take part in the respective cortices.

### 3.2. Heterogeneity of the Results

Table 1 reveals that the methodology and results of the studies appear quite heterogeneous. All but one study using vision-related acupoints indeed reported to have found activations, deactivations or both in some part of the visual cortex (Brodman areas 17, 18 and 19) [10]. For hearing-related acupoints the situation is similar, although there is only one positive and one negative result here. Cho et al. [22, 23] also published positive results on hearing-related acupoints in two books that will, however, not be covered here. While the variability of results is obvious between studies, Kong et al. [15] have also assessed the variability between and within subjects by measuring the test-retest reliability of acupuncture-fMRI. In their interesting approach six subjects underwent the same fMRI scanning procedure six times while receiving acupuncture. They found cortical activations observed under acupuncture stimulation to be much less consistent than those observed under the control task (finger-tapping).

Later in this review, we will give possible explanations for this variability as well as for other divergent results. Our hypothesis is that most of this variability is attributable to methodological problems that can even lead to false positive results.

We will cover a number of basic issues, that should be kept in mind while using fMRI to measure effects of acupuncture, namely how to interpret deactivations, which baseline to choose, how to control attention and why the choice of appropriate group statistics is so important.

## 4. Unsolved Problems of Acupuncture-fMRI Studies

### 4.1. The Issue of Deactivations

The frequent occurrence of deactivations in the studies analyzed here must be considered carefully. As Table 1 shows, three out of the seven studies using vision-related acupoints report deactivations in some part of the visual cortex. The origin of deactivations in fMRI studies has only recently been clarified [24]. From the mathematical point of view, deactivations are nothing more than negative correlations between the stimulation time course and the Blood Oxygenation Level Dependency (BOLD) signal. Let us assume that these changes in the BOLD signal are related to the stimulus. In this case there exist two possible explanations, namely, an actual reduction of neuronal activity caused by the stimulation (e.g., acupuncture) as compared to the baseline state or by neuronal activity that gets stronger in the baseline condition as compared to stimulation. Unfortunately these two possibilities cannot be distinguished, if one compares directly against baseline. Even if one compares two kinds of stimulation (e.g., real and placebo acupuncture), each of them has been measured directly against baseline. All this can only lead to valid results, if the baseline is well defined and stationary. However, the following section will show that, this is not the usual case.

### 4.2. Which Baseline to Use?

Almost all acupuncture-fMRI studies conducted so far have used “eyes closed” as a baseline for their measurements. That means, subjects were asked to close their eyes and to think of nothing special, while lying in the scanner. In some studies subjects were instructed to continuously focus on the stimulation, however, their actual level of attention was not controlled. The problematic aspect of this “low” baseline is, that the cortical activity associated with it is neither well defined nor stationary [25, 26]. As known for some time now, brain activity does not cease, when the subject is at rest. On the contrary there are several networks active when subjects are lying inside the scanner “doing nothing” [27, 28]. We will talk about these so called resting state networks (RSN) in more detail later. As Brandt has shown [29], mere closing of the eyes can lead to cortical activations, especially in visual and auditory cortices making its use in experiments on vision and hearing questionable.

#### 4.2.1. Uncontrolled Attention

Another problem when using “eyes closed” as a baseline is that the attention of subjects remains totally uncontrolled. Thus, they will probably focus on the acupuncture stimulation and pursue other cognitive tasks in the time between the stimulation blocks. These changes in attention can lead to cortical activations or deactivations as shown by a number of groups. One area that has often been shown to be activated or deactivated in attention changes is (again) the visual cortex [30–32]. Furthermore, a so called “default mode network” (DMN) [33] exists, that is usually active when subjects are doing nothing and thus often gets deactivated, when attention is drawn to some stimulation or cognitive task. This RSN comprises parts of the posterior parietal cortex, occipito-parietal junction, precuneus, posterior cingulate cortex and frontal pole. The idea that attention is responsible for some deactivations observed in acupuncture-fMRI studies is not new and has been put forth by several authors [16, 34]. The study of Kong et al. [16] is a particularly good example since the typical pattern of posterior areas of the DMN can be identified in the paper (Figure 2, top row), where deactivations under electro-acupuncture of a vision-related acupoint are reported. Consequently, the authors of this study consider the possibility that their results are caused by changes in attention rather than by the acupuncture stimulation itself.

Since two of the studies reviewed here [11, 13] used laser acupuncture stimulation, we must admit that at least for these studies, uncontrolled changes in attention can hardly explain the results. This is because in laser acupuncture the experimental design is double-blinded and the subject does not feel whether the laser is switched on or off. Thus its attention is not influenced by changing sensory inputs.

#### 4.2.2. Resting State

So far we have assumed that activations observed in acupuncture-fMRI studies were related to the stimulation in some way. However, there is also the opposite possibility of a purely random (anti-)correlation between the stimulation time course and the BOLD signal. As we have seen, the brain is not inactive, when the subject is at rest. On the contrary intrinsic brain activity called resting state is present at all times. This intrinsic activity of the brain at rest has recently drawn the attention of many scientists, especially in the field of fMRI. After PET experiments had shown task- independent deactivations, Raichle proposed the idea of a default mode of brain function [25, 33]. In recent years, many fMRI studies have been conducted and several additional RSNs have been identified [27, 28]. The frequency range of signal fluctuations in RSNs has been assessed by several groups finding values of *∼*0–0.1 Hz [35]. As several authors have pointed out, this frequency range considerably overlaps with repetition frequencies of standard block-design time courses used in fMRI [27, 35]. For example, a block length of 30 s plus rest blocks of equal length leads to a repetition frequency of *∼*0.017 Hz. In the case of an incidental phase correlation of intrinsic fluctuations with the time course of an fMRI experiment this can easily result in false positive activations or—in the case of an anti-correlation—to deactivations. One could argue that any cognitive task interrupts the resting state since the brain is no longer at rest. However, several studies have shown, that this is not the case [36, 37]. The results of Greicius et al. are especially relevant for acupuncture-fMRI, because they show that resting state activity is almost left unchanged in case of a passive, block-design, sensory task (such as manual needle acupuncture). Furthermore, the investigations by the groups of Litscher and Siedentopf were performed using laser needle stimulation, where the subject usually does not even notice if the laser is switched on or off. Because of the purely random character of the phase correlations explained here, two general statements can be made: firstly, using conservative thresholds only few subjects will show these false positive activations. Without further assumptions this effect could in principle explain results on single subject level as reported by the groups of Cho [3] and Litscher [13] but not group results. However, in the section on statistical group analyses we will show that some group statistics can easily turn strong single subject activations into significant group results. The second general statement is that there should be no preference of activations or deactivations, since correlations are as likely to happen as anti-correlations. This is more or less exactly, what we observe in acupuncture-fMRI studies. The amount of activations and deactivations is about the same on group level as well as between and within subject results (where these are reported). For example in the paper by Kong [15, Figures 5 and 6]; they found about the same amount of activations and deactivations. Possible cortical areas for such incidental (de-)activations theoretically include all those that are regularly found in resting state experiments. What is most important for the studies reviewed here is that three out of ten RSN as reported by Beckmann et al. [27] comprise either the visual or the auditory cortex, offering an alternative explanation for the (de-)activations found in these areas in acupuncture-fMRI experiments.

### 4.3. The Choice of Group Statistic

In fMRI two basic approaches are commonly used for group analyses: Fixed-effects (FFX) and random-effects analyses (RFX). While an RFX takes into account the between-subject-variance of the activations found on single subject level, FFX only considers within-subject-variance.  Table 1 reveals that most of the positive results on visual and auditory activations under acupuncture stem from studies that either used FFXs or no group statistics at all.

#### 4.3.1. FFX

It has long been known, that FFX suffers from a high susceptibility to outliers, that is, single subjects with strong activations, as it only accounts for within-subject variance [38]. Hence a single subject with a strong activation can in fact drive a whole FFX to produce significant results at group level that look as if the majority of subjects had shown this activation. This effect is especially relevant in the case of small sample sizes. As shown above there exists a mechanism that can produce such strong activations in single subjects, namely incidental phase correlations of resting state activity and the time course of the block-design. In fact, the use of FFX is deprecated today by most scientists exactly for this reason. A recent study by Smith et al. has quantified the effect of intrinsic brain activity on fMRI time series in single subject and FFX and has shown to be quite substantial [39].

#### 4.3.2. RFX

Only three out of nine studies covered in this review have used RFX and even these reported their results on an uncorrected level, that is, without applying any of the common correction methods for multiple comparisons. In fact, these are the only studies of those reviewed here that applied state-of-the-art statistics. A closer look at one of these RFX studies [12] reveals that only subjects showing visual activations on single subject level were included in the group analysis. This approach will almost certainly lead to positive group results. The two remaining RFX studies either yielded null results [17] or results that were interpreted by the authors as resulting from changes in attention [16].

## 5. Synopsis and Possible Solutions

While we are far from questioning the validity of using fMRI for the study of acupuncture effects, we think that visual and auditory activations in the studies reviewed here were probably not a direct result of acupuncture stimulation. We have seen that there exist several mechanisms other than acupuncture that can explain such activations. Using “eyes closed” as a baseline leaves attention of the subjects uncontrolled and can therefore lead to (de-)activations due to changes in attention as a result of needle stimulation. Mere closing of the eyes can also lead to activations in visual and auditory cortices. Furthermore, acupuncture may be too weak a stimulus to suppress resting state activity of the brain which may on its part lead to false positive (de-)activations in case of incidental phase correlations with the stimulus time course. RSN comprise both visual and auditory cortical areas.

Most of these problems can be overcome by using a different better controlled baseline. One possibility is a continuous attention task throughout the entire experiment. Attention may be controlled for, for example, by a changing visual stimulus, where the subject has to react to these changes by pressing a button. Although this is an untypical situation as compared to a standard therapeutic acupuncture treatment, we think the advantages clearly outweigh the disadvantages here. If the implementation of an attention task is impossible for any reason, researchers should at least familiarize themselves with the typical patterns of resting state activity.

As we have seen, the formerly widely used fixed-effect group analysis can easily turn strong activations of single subjects into positive group results even if the rest of the group did not show these activations. As a result this statistical method should not be used any more. Instead RFX should be the method of choice. These require a minimum of about 12 subjects; however, the aim should be to report results with a corrected threshold, which may require double the amount of subjects [40].

A final point to question the specificity of visual and auditory activations under acupuncture stimulation is that Parrish found hearing-related acupoints to activate visual cortical areas as well (see [14, Figures 1 and 2]). Furthermore, a large number of studies that did not use vision-related acupoints nevertheless found activations in visual cortical areas [9, 34, 41–53].

After pointing out the most important methodological problems of past acupuncture-fMRI studies, and showing their possible impact on study results, we would like to emphasize that our results do not contradict the existence of possible therapeutic effects of acupuncture. To name just one result, Litscher and colleagues have shown that laser needle stimulation of vision-related acupoints can increase blood flow in the ophthalmic artery, which is the largest supplying vessel of the eye [54]. This constitutes an alternative explanation for possible therapeutic effects of these acupoints. We hope that adopting the solutions offered here will lead to more appropriate studies and finally enable us to unravel the underlying mechanisms of acupuncture.

## Funding

F.B. would like to thank Manfred Köhnlechner Stiftung for funding support. C.H. was supported by BMBF (German Ministry of Education and Science).

## Figures and Tables

**Table 1:** Methodological and statistical details of acupuncture-fMRI studies reporting activations of visual or auditory cortices under stimulation of respective acupoints.

	*n*	Block length	Group statistics	Correction method	*P*-value	Stimulation method	Activations/ deactivations	(De-)activated Brodman areas	Acupoints used
Vision-related acupoints									
Cho et al. [3]	12	50s	None	n/a	n/a	Manual	Both	BA17, 18, 19^a^	BL60-67
Gareus et al. [10]^b^	8/6	60s	Other	n/a	*P* < .05	Manual	None	None	GB37
Siedentopf et al. [11]	10	40s	FFX	Cluster level	*P* < .05	Laser	Activations	BA18, 19	BL67
Li et al. [12]	18	20s	RFX^c^	None	*P* < .001	Manual/ electr.	Both	BA17, 18, 19	BL60, 65, 66, 67
Litscher et al. [13]	1	60s	None	Cluster level	*P* < .05	Laser	Activations	BA19	BL67, BL60
Parrish et al. [14]^d^	12	300s	FFX	None	n/a	Manual	Activations	BA17, 18, 19^a^	BL60
Kong et al. [15, 16]	6^e^	30s	RFX	None	*P* < .001	Electrical	Deactivations	BA17, 18, 19	GB37, BL60
Hearing-related acupoints									
Parrish et al. [14]^d^	12	300s	FFX	None	n/a	Manual	Activations	BA22, 41, 42^a^	KID3
Wesolowski et al. [17]^b^	20	30s	RFX	None	*P* < .001	Manual	None	None	GB43

^a^Derived from figures (not reported in article); ^b^Studies with negative results; ^c^Only subjects with activations on first level were included in second-level analysis; ^d^Study covers both visual and auditory points; ^e^Each subject was measured six times resulting in an effective *n* = 20 (according to the authors).
